# Effects of music-based group exercise in patients with acquired brain injury—a randomized controlled trial

**DOI:** 10.3389/fpsyg.2025.1650872

**Published:** 2026-02-03

**Authors:** Mareike Schrader, Tobias Strank, Annette Sterr, Stephan Bamborschke, Christian Dohle

**Affiliations:** 1 Department of Research, Fürst Donnersmarck Foundation of Berlin, Berlin, Germany; 2 Center for Stroke Research Berlin, Charité – Universitätsmedizin Berlin, Berlin, Germany; 3 P.A.N. Center for Post-Acute Neurorehabilitation, Berlin, Germany

**Keywords:** music therapy, cognition, mood, motivation, group therapy, neurorehabilitation, acquired brain injury, stroke

## Abstract

**Background:**

Cognitive impairment following acquired brain injury (ABI) is common. In this study, we investigated whether music-based group exercise (MBGE) is superior to standard therapy (ST) in improving cognitive deficits. In addition, motivational aspects were investigated. The trial has been registered with the German Register for Clinical Studies (DRKS00025566).

**Method:**

In the experimental group of this randomized controlled trial, sensors were attached to conventional rehabilitation exercise equipment that used software (Jymmin^®^) to convert physical activity into good-sounding music. Three patients exercised at the same time and produced a piece of music together. The control group trained individually using the same machines, but without the music-producing sensors. The training schedule comprised four weeks, with three 30-min training session per week, respectively. The Montreal Cognitive Assessment, four subtests of the Test Battery for Attention, the Bayer Activities of Daily Living Scale, the Multidimensional Mood Questionnaire and a questionnaire on motivational aspects and preferred training constellation were carried out before (T1), after the intervention (T2) and 3 months later (T3).

**Results:**

35 patients (MBGE: *n* = 17; ST: *n* = 18) completed the intervention and were included in the analysis. Both groups benefited from the exercise but no significant differences were found between the groups neither in the cognitive assessments, activities of daily living (ADL) nor mood. Group constellation was associated with greater enjoyment, whereas individual therapy was associated with subjectively perceived better concentration and more intensive training.

**Discussion:**

MBGE was not found to be superior to ST regarding cognition, ADL and mood. Both approaches demonstrated similar potential to positively influence these areas. Generally, individual preferences for group or single constellation should be considered. Further studies are needed to strengthen the evidence base for music-assisted therapy that addresses cognition and mood in people with ABI.

**Clinical trial registration:**

https://drks.de/search/de/trial/DRKS00025566, identifier DRKS00025566.

## Introduction

Acquired brain injuries (ABI) refer to damage or injury to the brain after birth that occurs either traumatically, such as by a traumatic brain injury (TBI), or non-traumatically, such as by a stroke, tumor or cerebral anoxia (Goldman et al., 2022). Cognitive difficulties are common in ABI (Jokinen et al., 2015; Lennon et al., 2023), and often remain a major problem for patients in the long-term (Barker-Collo et al., 2010). Cognitive skills include among others attention, memory, perception and planning. In individuals with TBI, the domains of processing speed, executive functioning, and memory are predominantly affected (Svingos et al., 2019; Sigurdardottir et al., 2020). Cognitive impairments primarily occur in association with cortical brain injuries, such as damage to the prefrontal cortex or the temporal lobe following a TBI (Cristofori and Levin, 2015). For stroke, different studies have reported a prevalence of cognitive impairment ranging from 20% to 80%, varying between countries (Sun et al., 2014). More than half of the stroke patients with attention control problems report difficulty with concentrating, forgetfulness, rapid fatigue, and dual task demands (Hochstenbach et al., 2005). These skills are important for coping with everyday life and for living as independently as possible. Attentional and visuospatial skills are also considered to be important predictors of treatment efficacy with regard to activities of daily living (Watson et al., 2020). Another frequent complication is the presence of mood disorders, which commonly co-occur with cognitive impairments (Murphy et al., 2024), with the most common complication being depression, usually defined as post-stroke depression. The incidence of depression ranges between 11% and 41% following stroke (Guo et al., 2022), and is approximately 13% among patients with TBI (Dehbozorgi et al., 2024). Patients with post-stroke depression have a lower rate of recovery from functional impairment than non-depressed patients (Hadidi et al., 2009).

Not only do cognitive and mood difficulties coexist alongside each other, their therapy is notoriously difficult. The therapy of cognitive impairments after ABI requires an individualized, interdisciplinary approach that includes neuropsychological training programs, compensatory strategies, as well as occupational, physical, and speech therapy interventions. To reduce cognitive deficits, computer-assisted therapy methods that train attention in specific situations similar to everyday life are recommended (Bogdanova et al., 2016; Cicerone et al., 2019; Shahmoradi et al., 2022; Nie et al., 2022). However, not all patients benefit from this type of training and are motivated to use digital cognitive training tools. Mood disorders are mainly treated pharmacologically (Towfighi et al., 2017), but there is not always a willingness to take these drugs (Sansone and Sansone, 2012). Other forms of therapy are therefore needed for both conditions.

Music therapy has a wide range of applications in the field of neurorehabilitation (Sihvonen et al., 2017). For example, music therapy is used in early neurological rehabilitation to enable non-verbal communication with severely affected patients (Schönebaum and Bamborschke, 2003), and in gait therapy is used to provide rhythmic acoustic stimulation (Yoo and Kim, 2016). The realization that timing and sequencing are also crucial to cognitive abilities (Conway et al., 2009) has been a driver for research into the potential of music for cognitive rehabilitation (Thaut et al., 2015). There is good evidence that music training and learning has an impact on functional and structural changes in the brain, particularly in motor regions (Münte et al., 2002; Altenmüller and Schlaug, 2015). Listening to music has been shown to mediate the recovery of verbal memory and attention after stroke (Särkämö et al., 2008), and to improve working memory in patients with moderate dementia (Särkämö et al., 2015). However, the effect of music-based therapies on cognition in people with ABI has not yet been clearly demonstrated (Magee et al., 2017; Loetscher et al., 2019; Rajendran and Summa-Chadwick, 2022). Similar to music, physical exercise has also been shown to enhance cognitive functioning in neurological patients (Cumming et al., 2012; Oberlin et al., 2017), although the evidence in this field remains inconclusive (Saunders et al., 2020). Beyond cognitive outcomes, music therapy can exert positive effects on mood (Särkämö et al., 2008; Chu et al., 2014; Dingle et al., 2021; Raglio et al., 2015). It can modulate activity in brain structures involved in, among other things, pleasant emotions (Koelsch et al., 2006), and therefore has the potential to be beneficial in the treatment of psychiatric and neurological disorders. Training in a group setting also has the potential to positively affect mood (Dingle et al., 2021). Group therapy is an opportunity for peoples with disabilities for friendship and social interaction (Graham et al., 2008). Making music together constitutes an enjoyable social activity that enhances mood (Pohl et al., 2020; Dingle et al., 2021) and strengthens social interaction within the group (Dingle et al., 2021; Fritz et al., 2015; Pohl et al., 2018).

With the present study, we report the effect of “Jymmin^®^” which combines these approaches, defined as the combination of gym (fitness room) and jamming (making music together). It uses sensitive sensors attached to conventional exercise equipment to translate the movement of arms and legs into harmonious rhythms and melodies via specialized software (Fritz et al., 2013). The active control and co-creation of music through one’s own movements during training is defined as musical agency. The intervention is carried out in a small group of three people who can act as a small “band.” “Jymmin^®^” has demonstrated benefits for short-term memory in patients with dementia (Strong et al., 2022), but its impact on cognitive skills in people with ABI is unclear. We propose that performing exercises that generate sound will require increased attentional control, and that both physical activity and cognitive performance have a positive effect on activities of daily living (ADLs). Furthermore, we anticipate that group exercise will be more enjoyable, thereby improving participants’ mood as a related study with healthy participants showed (Fritz et al., 2013). The aim of the following trial was to investigate whether music-based group exercise (MBGE) is superior in improving cognitive deficits, activity of daily living (ADL) and mood compared with standard therapy (ST) consisting of the same exercise in a single setting without music.

## Materials and methods

### Trial design and setting

The present study is an assessor-blinded, parallel-group randomized controlled trial conducted from August 2021 to June 2023 in a long-term neurorehabilitation facility for patients with ABI in Germany (Center for Post-Akute Neurorehabilitation in Berlin) (Schrader et al., 2024). The study protocol was approved by the local ethics committee (Ärztekammer Berlin) and registered with the German Register for Clinical Studies (DRKS00025566). Written informed consent was obtained from all participants prior to participation.

### Inclusion criteria and randomisation

All patients receiving treatment at the center were screened for eligibility using the following inclusion criteria: (a) ABI, including stroke, TBI, hypoxic brain damage, or other causes; patients with degenerative or progressive conditions such as Parkinson’s disease were excluded (b) age ≥ 18 years, (c) ability to follow therapist instructions, (d) cardiac capacity to perform 30 min of physical training (including breaks). Exclusion criteria were (e) severe aphasia, (f) severe hearing impairment and (g) acute rheumatism.

Group allocation was performed in groups of three patients each, recruited consecutively. As the study was conducted during the COVID pandemic, it was not possible to form groups across wards. Instead, the three participants forming a training group were recruited from the same shared flat (unit; similar to a ward in a hospital). The groups were randomly allocated to one of the two conditions (MBGE or ST). Group randomisation took place in blocks of 4, using a pre-defined list available only to a person not involved in the study (secretary’s office).

### Intervention

Patients received either MBGE or ST in 30-min sessions, three times a week, for four consecutive weeks. Patients were included in the analysis if they participated in at least nine sessions, resulting in a minimum training time of 270 min overall. The same physical exercises were used in both groups, depending on the patient’s ability: (a) pulling down an expander; (b) cycling on a motor assisted leg or arm trainer or cycling on an ergometer; (c) whole body exercise on a recumbent cross trainer (NuStep^®^). These devices are an integral part of the standard rehabilitation programme. During one session, each patient exercised on one machine.

The Jymmin^®^ technology in the MBGE group merges musically expressive performance with physical exercise. Sensors were attached on the exercise devices or directly on the body to measure precise movement amplitudes and speeds and convert these signals into harmonious rhythms and melodies using specially developed software. Each patient is assigned to their own sound/instrument (e.g., drums, trumpet, guitar) via the software on the tablet. All three patients create a piece of music together, like jamming in a band. The instrument can thereby be controlled through changes in movement, e.g., by moving faster to increase the volume. At the beginning of each session the system was calibrated to the individual level of ability of each patient. Three exercise stations were set up in close proximity to each other in the MBGE group, forming a semi-circle so that everyone could see each other. During the session, the therapist controlled the software and guided the participants like a conductor. For example, the therapist indicates whether all three should play simultaneously or if one takes a solo while the others briefly pause or play more softly to place their sound in the background. Therefore, patients needed to be attentive and listen to the others while performing their own tasks. In the ST group, each patient trained individually on their own exercise machine without the music component as described above (except of radio music played in the background).

In addition to the intervention, participants received conventional therapies at the rehabilitation facility, totaling approximately 7.7 h per week in physiotherapy, occupational therapy, speech therapy, and neuropsychology (Schrader et al., 2024). These therapies can also have an impact on cognition, mood, and physical health.

### Outcome parameters

#### Montreal Cognitive Assessment (MoCA)

The primary outcome measure was the MoCA (Nasreddine et al., 2005), a well-established assessment tool for mild cognitive impairment. The MoCA was selected particularly because it is time-efficient and provides a good overview of different cognitive domains by assessing seven domains through a total of 11 tasks. The domains are: (a) executive and visuospatial function, (b) naming, (c) attention, (d) language, (e) abstraction, (f) delayed recall, and (g) orientation. The MoCA has a maximum score of 30 points. The cut-off score for cognitive impairment is less than 26 points. The validity and reliability of the MoCA is considered to be high (Nasreddine et al., 2005). The items were administered equally to all participants, including patients with light to moderate aphasia or visual neglect. It cannot be ruled out that this did not create equal conditions for solving the tasks. During drawing, the paper was held steady if needed so that even patients with hemiparesis had the opportunity to draw.

#### Test of Attentional Performance (TAP)

The TAP (Zimmermann and Fimm, 2012) is a computerized multidimensional test of attentional disorders consisting of 12 subtests. All subtests have standardized instructions that are presented on the screen before the test is administered. The TAP selects simple response paradigms in which selective responses can be made to easily discriminable, non-verbal stimuli by pressing a button under different conditions. The performance criteria are reaction time and accuracy (number of incorrect responses and omissions). In this study, the subtests Alertness, Divided Attention, Flexibility and Go Nogo were used (Zimmermann and Fimm, 2012). Prior to the start of the study, a digitally randomized order of subtests was created and maintained for each participant at all three test times.

#### Multidimensional Mood Questionnaire (MDMQ)

The MDMQ (Steyer et al., 1997) consists of a 24-item long form and a 12-item short form. The long form was used at the three main timepoints, and the short form was used after each session to capture participant’s immediate mood after training. The MDMQ measures three bipolar dimensions of current mental state: good/bad mood (GB), alertness/tiredness (AT) and calmness/agitation (CA). The items consist of simple adjectives such as “tired,” “well” and are scored on a five-point response scale with endpoints 1 (not at all) and 5 (very much). The “negative” items need to be recoded to positive before summation. Scores range between 8 and 40 points for the long version and between 4 and 20 points for the short version. A high value is an indication of positive mood, alertness or calmness. The MDMQ has been found to be highly reliable and classified as valid (Steyer et al., 1997).

#### Bayer Activities of Daily Living Scale (B-ADL)

The B-ADL (Hindmarch et al., 1998) is an external assessment tool to measure impairment in ADL in older patients with cognitive impairment. It uses 25 items to classify everyday problems on a ten-point scale. Item scores range from 1 (never difficulties) to 10 (always difficulties). The assessment is based on the information provided by the staff providing daily support, who should indicate how often the person has difficulty with the required ADL. Items that are not currently performed can be marked as “not applicable.” The category “don’t know” is selected when insufficient information is available. The total score is divided by the actual number of items that received a score between 1 and 10. Scores between 1.0 and 2.0 indicate no difficulty in coping with everyday life. Slight difficulties are indicated by scores between 2.1 and 5.0, and severe difficulties by scores between 5.1 and 10.

Data, such as diagnosis or the time since onset, was extracted from the medical reports. All outcome variables were measured before the intervention (pre = T1), after the 4-week intervention (post = T2) and three months after T2 (follow-up = T3). Additionally, the MDMQ short form was measured after each training session (TR1 up to TR12). After T2, a short self-designed questionnaire was administered, assessing motivation and preference regarding the training group (individual or group therapy). All assessments were conducted according to the instructions in the test manuals, regardless of whether a speech impairment, neglect, or functional deficits were present, in order to ensure comparability across the different testing time points.

### Statistical analyses

Statistical analyses (per-protocol) were performed using SPSS Statistics 28.0, and graphs were generated with Excel. Descriptive statistics comprised means (M) and standard deviations (SD), medians (MD), interquartile ranges (IQR), and frequencies (*N*; %) for continuous and categorical variables, respectively.

A multilevel model (MLM) was calculated, comprising a macro-level factor (patients) and a micro-level factor (repeated measures) for outcome parameters where the residuals were approximately normally distributed (MoCA, TAP Alertness, B-ADL). For data not meeting the MLM assumptions, the non-parametric methods Mann–Whitney-U-test (MWU) and Friedman test were used (TAP Flexibility, Divided Attention, Go Nogo, MDBF). For readability, analyses for all outcomes are presented non-parametrically; MLM analyses are provided in the Supplementary material. *Post-hoc* tests (Dumm–Bonferroni) were calculated to determine the source of the significant differences between time points.

Depending on the result of the questionnaire at T2, an additional *post-hoc* analysis was conducted that analyzed the data separately for those patients who had trained in their preferred group (yes/no). The self-designed questionnaire was analyzed descriptively.

## Results

### Study population

**FIGURE 1:**
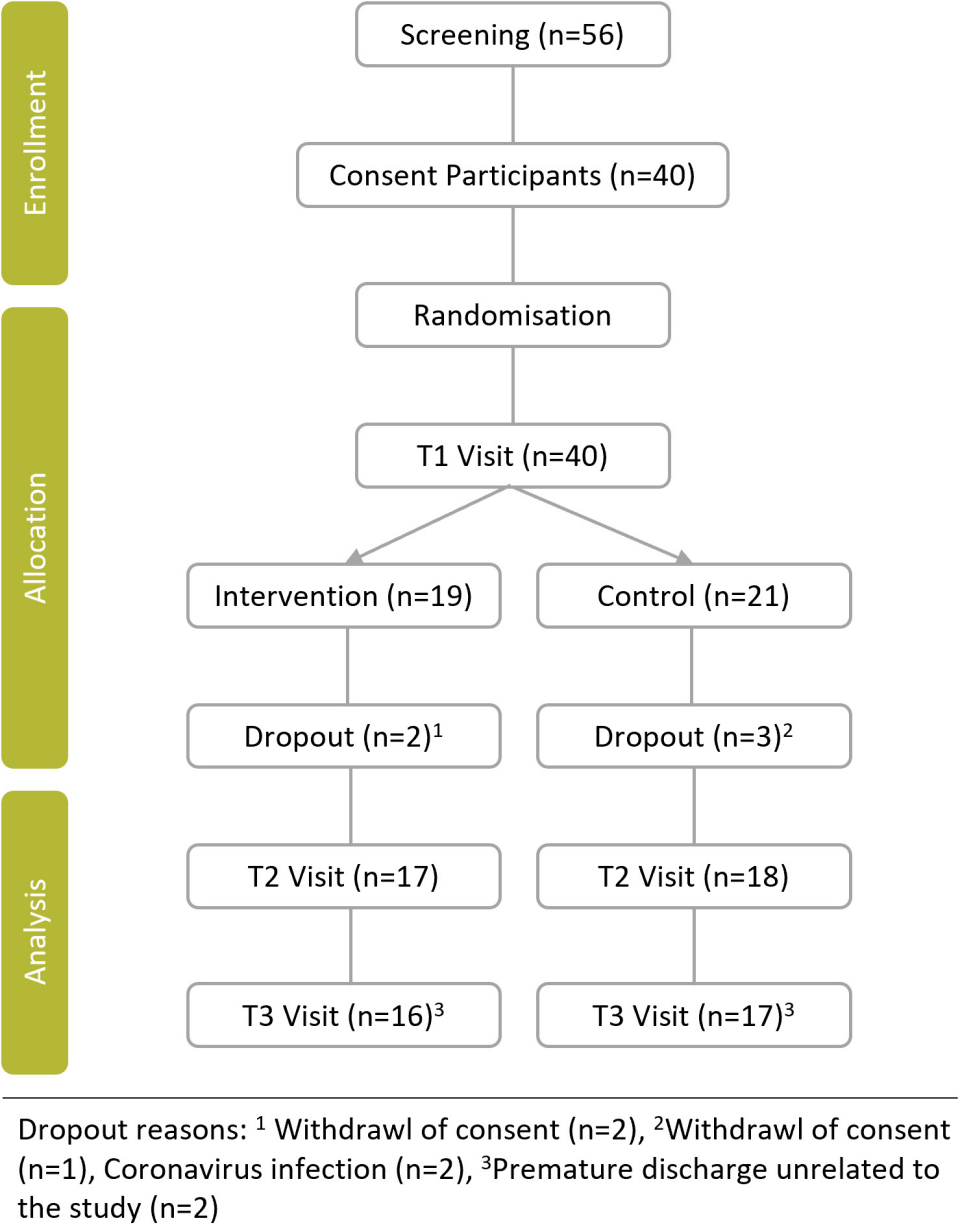
Flow chart diagram.

During the study period, 40 patients agreed to participate. A total of 35 patients completed the protocol and were included in the per-protocol analysis (Figure 1).

Demographic data at T1 are shown in Table 1. In both groups, more than 70% had suffered a stroke. Median time since the event was 13.5 months in MBGE and 11.5 month in ST group. None of the demographic parameters showed significant differences between the groups, although the participants in the ST group were slightly younger, but not significantly so, with a median age of 43 years (range: 24–57 years), compared to those in the MBGE group, who had a median age of 51 years (range: 21–64 years).

**TABLE 1:** Demographic data at T1.

*N* = 35		Intervention (*N* = 17)	Control (*N* = 18)	*p*-value
Age (years) Md (IQR)		51 (42; 61)	43 (38.3; 53.5)	0.053 (MWU)
Gender N (%)	Female	6 (35.3)	8 (44.4)	0.594 (X^2^)
	Male	11 (64.7)	10 (55.6)	
School education N (%)	>12 years	15 (88.2)	14 (77.8)	0.427 (X^2^)
	<12 years	2 (11.8)	4 (22.2)	
Diagnosis N (%)	TBI	3 (17.6)	4 (22.2)	0.445 (X^2^)
	Stroke	12 (70.6)	13 (72.2)	
	Other^1^	2 (11.8)	1 (5.6)	
Time since onset (month) Md (IQR)		13.5 (11.2; 16.6)	11.5 (8.1; 13.7)	0.318 (MWU)
Hemisphere affected N (%)	Right	10 (58.8)	8 (44.4)	0.265 (X^2)^
	Left	5 (29.4)	5 (27.8)	
	Both	2 (11.8)	5 (27.8)	
Neglect N (%)		4 (23.5)	5 (27.8)	0.782 (X^2^)
Motricity Index Md (IQR)	Arm	56 (14.5; 84.5)	35 (1; 79.8)	0.568 (X^2^)
	Leg	48 (29; 70)	52.5 (35.8; 85)	0.351 (X^2^)
FAC Md (IQR)		2 (1; 4)	4 (2; 4,3)	0.424 (MWU)

TBI, traumatic brain injury; FAC, functional ambulation category; Md, median; IQR, interquartile range; MWU, Mann–Whitney-U-test; X^2^, Chi-quadrat-test;

^1^Hypoxic brain injury and central pontine myelinolysis.

### Effects on cognitive abilities (MoCA, TAP)

**FIGURE 2:**
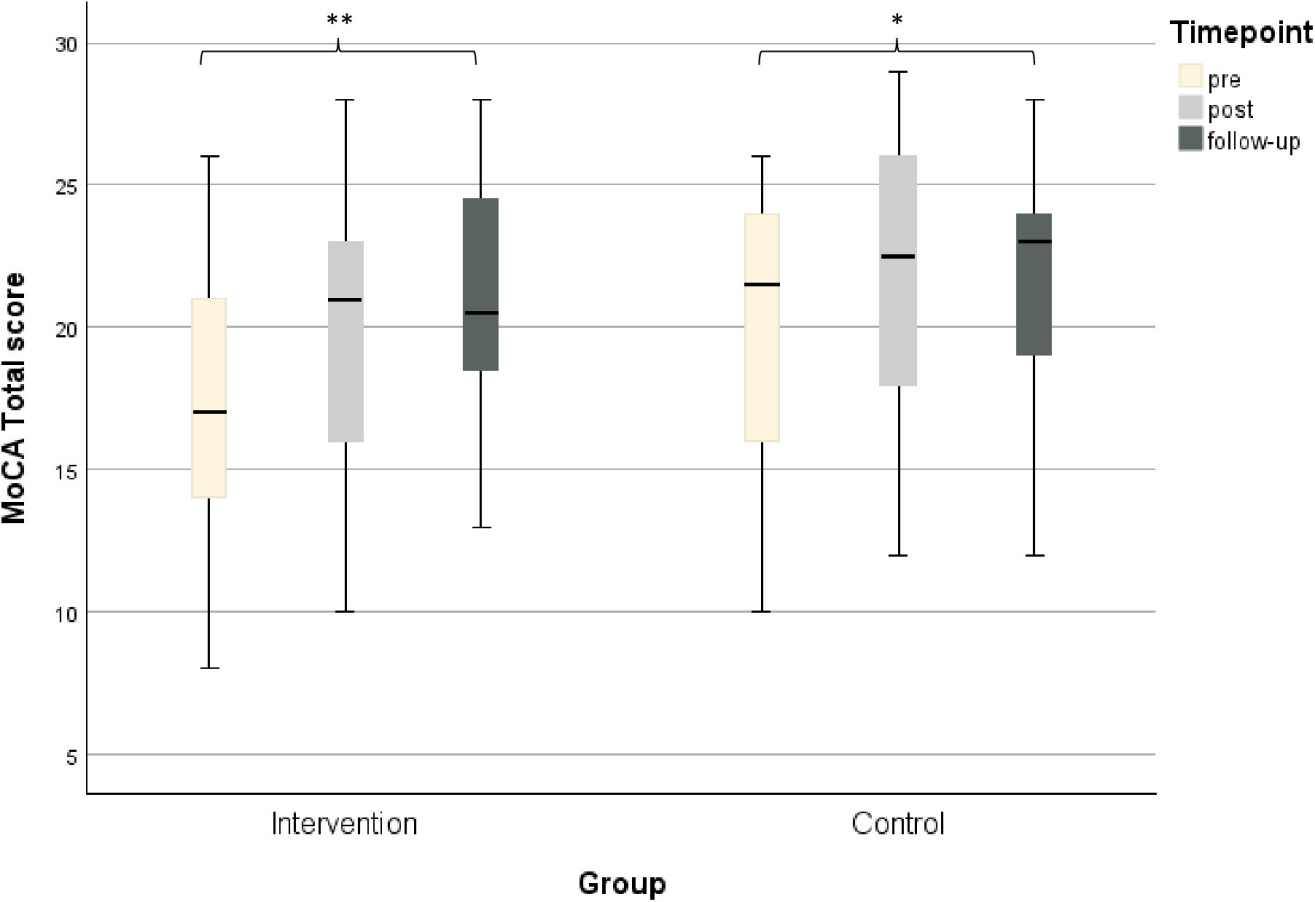
Median change scores and interquartile range within groups for the MoCA (*n* = 35); *p < 0.05; **p < 0.001.

Comparisons within groups of the MoCA revealed significant differences in timepoints for the MBGE group (Friedman test, *p* < 0.001) as well as for the ST group (Friedman test, *p* = 0.037) (Table 2). In both groups cognitive performance in the MoCA improved. *Post-hoc* tests showed that the time points pre and follow-up differ significantly for the MBGE group (Dunn–Bonferroni, *z* = −1.344, *p*_*adjusted*_ < 0.001) as well as for the ST group (*z* = −0.824, *p*_*adjusted*_ = 0.049) (Figure 2).

**TABLE 2:** Within group comparison related to time points.

Assessment	Score	Group	Timepoint; Md [IQR]			*P*
			Pre	Post	Follow-up	Friedman test
MoCA	Total score	I	16.5 [13.3; 22.5]	21 [15.3; 23.8]	20.5 [18.3; 25.3]	< 0.001**
		C	21 [15.5; 24.5]	22 [16.5; 25]	23 [17.5; 24.5]	0.037*
TAP Alertness	Respond speed (ms) with signal	I	391 [265; 430]	356 [260; 441]	334 [274; 401]	0.174
		C	297 [278; 379]	308 [285; 363]	290 [272; 343]	0.067
	Respond speed (ms) without signal	I	407 [276; 510]	345 [283; 470]	353 [277; 472]	0.012*
		C	333 [298; 376]	360 [313; 395]	337 [287; 385]	0.257
TAP Flexibility	Respond speed (ms)	I	964 [810; 1,620]	897 [740; 1,285]	867 [740; 1,338]	0.285
		C	992 [712; 1,482]	890 [677; 1,365]	835 [673; 1,353]	0.943
	Number of errors	I	2 [0; 2.8]	1 [0; 2]	0.5 [0; 2.8]	0.452
		C	1 [0; 3]	1 [0; 2]	0 [0; 1]	0.311
TAP Divided Attention	Omitted hits auditive	I	6 [1.3; 8]	8 [1; 13]	6 [1; 14.5]	0.341
		C	4.5 [1; 9.8]	3.5 [1.3; 8.5]	3 [1; 6.3]	0.759
	Omitted hits visual	I	2 [0; 10.8]	1.5 [0; 10.5]	0 [0; 5.5]	0.103
		C	0 [0; 6.5]	1 [0; 12.5]	0 [0; 5]	0.972
	Omitted hits total	I	8.5 [2.5; 18.5]	12.5 [1; 21.5]	12 [1; 19.8]	0.595
		C	8 [2.8; 12.3]	5.5 [2; 17.8]	4 [1; 11.5]	0.607
TAP Go Nogo	Respond speed (ms)	I	563 [499; 617]	520 [480; 682]	477 [432; 640]	0.549
		C	550 [447; 632]	501 [445; 572]	460 [440; 558]	0.080
	Number of errors	I	0 [0; 2]	1 [0; 1]	0 [0; 3]	0.439
		C	1 [0; 2.5]	1 [0; 1]	0 [0; 1]	0.412
MDMQ	Good/bad mood (total score)	I	31.5 [24; 37.8]	35 [27.5; 37]	35 [25.5; 38.8]	0.340
		C	34 [29.5; 38]	38 [28; 39.5]	36 [28; 37.5]	0.016*
	Alertness/tiredness (total score)	I	29 [20.3; 34.5]	33.5 [25.3; 36.8]	32.5 [26.8; 37.8]	0.077
		C	35 [27; 37.5]	37 [31.5; 39]	32 [23; 39]	0.219
	Calmness/agitation (total score)	I	27.5 [26; 32.8]	35.5 [32.3; 36]	31 [29; 37]	0.010*
		C	35 [31.5; 37.5]	37 [30.5; 40]	37 [29.5; 40]	0.021*
B-ADL	Total score	I	5.6 [4.2; 7.4]	4.6 [3.6; 6.9]	4.6 [3.4; 5.9]	0.035*
		C	5.3 [4.1; 7]	4.5 [3.6; 6.4]	4.4 [3.6; 5.7]	0.002*

MoCA: Montreal Cognitive Assessment; TAP: Test of Attentional Performance; MDMQ: Multidimensional Mood Questionnaire; B-ADL: Bayer Activities of Daily Living Scale; ms: milliseconds; Md: median; IQR: interquartile range; I: intervention; C: control;

*p < 0.05;

**p < 0.001; *N* = 33 (two lost to follow-up).

Comparisons within groups revealed also a significant difference for the TAP Alertness respond speed without signal in the MBGE group (Friedman test, *p* = 0.012) (Table 2) for the time points pre and follow-up (Dunn–Bonferroni, *Z* = 0.969, *p*_*adjusted*_ = 0.018). For all of the other time intervals not mentioned above, no significant differences were observed within the groups.

The between group comparison showed no significant difference for the MoCA and any of the other cognitive assessments in the MWU (Table 3). As the residuals for the MoCA and TAP Alertness were approximately normally distributed, additionally an MLM was computed. The MLM revealed significant effects of “time” on MoCA scores and TAP Alertness response speed with signal, but, consistent with the non-parametric analysis, no significant effects of “group” were observed (Supplementary Table 1).

**TABLE 3:** Between group comparison related to different time points.

Assessment	Score	Timepoint	Intervention (*n* = 17)	Control (*n* = 18)	*p* (MWU)
			Md (IQR)	Md (IQR)	
MoCA	Total score	Pre	17 [13.5; 22]	21.5 [15.8; 24.3]	0.096
		Post	21 [15.5; 23.5]	22.5 [17.3; 26]	0.351
		Follow-up	20.5 [18.3; 25.3]	23 [17.5; 24.5]	0.631
TAP Alertness	Respond speed (ms) with signal	Pre	389 [271; 430]	301 [281; 376]	0.173
		Post	364 [263; 441]	310 [285; 361]	0.660
		Follow-up	334 [274; 401]	290 [272; 343]	0.465
	Respond speed (ms) without signal	Pre	404 [277; 505]	343 [298; 395]	0.232
		Post	343 [287; 455]	366 [318; 389]	0.660
		Follow-up	353 [277; 472]	337 [287; 385]	0.683
TAP Flexibility	Respond speed (ms)	Pre	983 [800; 1,339]	977 [669; 1,682]	0.568
		Post	872 [784; 1,185]	863 [683; 1,350]	0.909
		Follow-up	835 [720; 1,344]	950 [662; 1,382]	0.873
	Number of errors	Pre	1 [0; 2.5]	2 [0; 4]	0.987
		Post	0 [0; 1]	1 [0; 2]	0.858
		Follow-up	0 [0; 1]	0.5 [0; 1.8]	0.465
TAP Divided Attention	Omitted hits Auditive	Pre	5 [1; 8]	4 [1; 95]	0.946
		Post	8 [0.5; 13]	3 [1.5; 8]	0.610
		Follow-up	6 [1; 14.5]	3 [1; 6.3]	0.445
	Omitted hits visual	Pre	2 [0; 10.5]	0 [0; 6]	0.563
		Post	1 [0; 9]	1 [0; 10]	0.919
		Follow-up	0 [0; 5.5]	0 [0; 5]	0.838
	Omitted hits total	Pre	8 [1.5; 18]	7 [3; 11.5]	0.838
		Post	11 [0.5; 21]	6 [2; 15.5]	0.865
		Follow-up	12 [1; 19.8]	4 [1; 11.5]	0.642
TAP Go Nogo	Respond speed (ms)	Pre	558 [499; 617]	544 [442; 624]	0.484
		Post	516 [483; 680]	496 [415; 568]	0.266
		Follow-up	477 [432; 640]	460 [440; 558]	0.710
	Number of errors	Pre	0 [0; 2]	1 [0; 2.3]	0.621
		Post	1 [0; 1]	1 [0; 1]	0.670
		Follow-up	0 [0; 3]	0 [0; 1]	0.433
MDMQ	Good/bad mood	Pre	30 [23; 37.5]	34 [27.5; 38]	0.386
		Post	35 [27; 37]	36.5 [28; 39.3]	0.303
		Follow-up	35 [25.5; 38.8]	36 [28; 37.5]	0.986
	Alertness/tiredness	Pre	26 [20.5; 34]	34.5 [25.8; 37.3]	0.096
		Post	34 [25.5; 36.5]	36.5 [32.3; 39]	0.303
		Follow-up	32.5 [26.8; 37.8]	32 [23; 39]	1
	Calmness/agitation	Pre	27 [25.5; 32.5]	35 [29; 37.3]	0.077
		Post	35 [32.5; 36]	37 [30.8; 40]	0.153
		Follow-up	31 [29; 37]	37 [29.5; 40]	0.136
B-ADL	Total score	Pre	5.8 [4.2; 7.4]	5.6 [4.2; 6.8]	0.987
		Post	4.6 [3.7; 6.8]	4.5 [3.8; 6.5]	0.732
		Follow-up	4.6 [3.4; 5.9]	4.4 [3.6; 5.7]	0.817

MoCA, Montreal Cognitive Assessment; TAP, Test of Attentional Performance; MDMQ, Multidimensional Mood Questionnaire; B-ADL, Bayer Activities of Daily Living Scale; ms, milliseconds; Md, median; IQR, interquartile range; MWU, Mann–Whitney-U-test.

Since the age differences between the groups at T1 were borderline significant in the MWU (*p* = 0.053) an additional MLM with age as covariate for the primary outcome MoCA and the TAP Alertness, was calculated. This analysis did not yield significant results between groups (Supplementary Table 2).

### Effects on mood/mental state (MDMQ)

In the long version of the MDMQ comparisons within groups revealed a significant difference for timepoints for the subscale “*good/bad mood*” for the ST group (Friedman test, *p* = 0.016) (Table 2). *Post-hoc* test showed that the timepoint post and follow-up differ significantly (Dunn–Bonferroni, *z* = 0.912, *p*_*adjusted*_ 0.024). The subscale “*calmness/agitation*” revealed a significant difference for timepoints for the MBGE group (Friedman test, *p* = 0.010) and the ST group (Friedman test, *p* = 0.021) (Table 2). *Post-hoc* tests showed that the time points pre and post differ significantly (Dunn–Bonferroni, MBGE: *z* = −1.0, *p*_*adjusted*_ 0.014; ST: *z* = 0.853, *p*_*adjusted*_ 0.039). No significant differences were observed at any of the other time intervals not mentioned above within the groups.

The MDMQ showed no significant differences between group on any of the MDMQ dimensions in the MWU (Table 3).

For the short version of the MDMQ administered after each session, a mixed ANOVA could not be calculated as there was no normal distribution at most of the 12 time points. The mood of the patients in both groups was in the upper part of the range (Supplementary Figure 1). There were no significant differences in the MWU test at any of the training time points.

### Effects on activities of daily living (B-ADL)

Participants in both groups improved their B-ADL abilities over time. The comparisons within groups revealed a significant difference for the MBGE group (Friedman test, *p* = 0.035) as well as the ST group (Friedman test, *p* = 0.002) (Table 2). *Post-hoc* test for the MBGE group showed that the timepoint pre and follow-up differed significantly (Dunn–Bonferroni, *z* = 0.906, *p*_*adjusted*_ 0.031), while differences in the ST group were both significant pre and post (Dunn–Bonferroni, *z* = 1, *p*_*adjusted*_ 0.011) as well as pre and follow-up (Dunn–Bonferroni, *z* = 0.1.1, *p*_*adjusted*_ 0.003). No significant differences were observed at any of the other time points not mentioned above within the groups.

The B-ADL showed no significant differences between the groups in the MWU (Table 3). As the residuals for the B-ADL were approximately normally distributed, an additional MLM was computed. This analysis confirmed the absence of significant group effects already found through the non-parametric analysis (Supplementary Table 1).

### Effects of personal preferences

**FIGURE 3:**
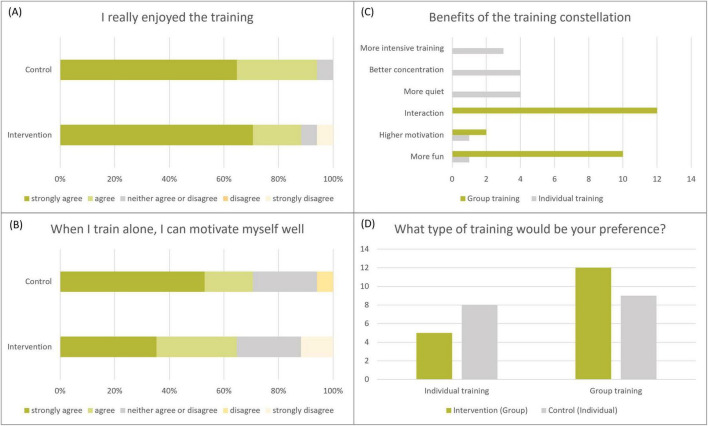
**(A–D)** Motivation questionnaire.

The results of the questionnaire on motivational aspects and preferred training constellation are shown in Figures 3A–D.

Participants in both groups found their training predominantly enjoyable (Figure 3A). Participants in the ST group rated their motivation for training alone slightly higher than participants in the MBGE group (Figure 3B). Six categories were identified as reasons for the benefits of the training constellation (Figure 3C). The main reason given for preferring group exercise is that training in a group was regarded to be more fun and motivating. Interaction with others also played a major role. “*It’s more fun in a group, you motivate each other, you exchange ideas, you compete with the others*” (J34) summarized one of the participants. The advantage of an individual training session was claimed to be able to focus more on yourself. The following statement was made by a participant in the MBGE group “*If I want to make progress, I should train on my own, but it has still been a lot of fun*” (J31).

To explore the role of personal preference, a separate analysis was conducted for those whose preference and group allocation were aligned (*N* = 20), and for those whose preference and group allocation were misaligned (*N* = 14) (Figure 3D). This analysis revealed no significant differences in any of the outcome parameters except for the subscale “*good/bad mood*” which was significantly different at follow-up. At this timepoint those who were in their preferred constellation were in a better mood than those who were not (MWU, *z* = −1.95, *p* = 0.050) (Supplementary Table 3).

## Discussion

The study investigated the impact of music-based group exercise (MBGE) on cognitive performance, ADL and mood compared to a standard therapy (ST), in which patients performed exercises without creating music. We hypothesized that performing exercise with producing musical feedback would place a greater demand on the attention system and hence result in improvements in the MoCA and TAP. We further reasoned that exercising in a group and the musical feedback would be more enjoyable and hence improve mood as well as ADL abilities. In contrast to our expectations MBGE was not found to be superior to ST on cognition, mood or ADLs. However, both approaches provided improvements in the MoCA, ADLs and mood with regard to feelings of inner calm and serenity. Data of the questionnaire indicated that group therapy was associated with greater enjoyment, while individual therapy was associated with better concentration and more intensive training.

### Effects on cognitive functions

While these results are partly unexpected they are in line with some findings of the literature. For example, a crossover trial using the same music-based group training in older adults with dementia also showed limited effects for cognitive domains, with only short-term memory showing an advantage over the control group (Strong et al., 2022). In our study short term memory was not measured specifically. Moreover, our study was conducted in ABI patients who were still in intensive rehabilitation, being engaged in multiple additional therapy sessions throughout the day (Schrader et al., 2024). In contrast, Strong’s study only tested individuals with dementia, a different neurological condition, who lived in a nursing home and most likely did not receive any other intervention. The combination of these two factors is likely to explain the discrepancy between our and their findings. Likewise, no differences were reported in a study of Parkinson’s patients in which a comparable musical approach — the Ronny Gardener Method — was used (Pohl et al., 2020). This method combines audio, visual, tactile, and kinetic energy with rhythm, music and sound/movement codes. Here, the potential benefits of the treatment were compared to a control group that received no treatment. However, there were no changes visible on the MoCA between the groups either. Unlike the study by Pohl, patients in our study showed significant within-group differences in the MoCA. This may be related to the fact that patients in Pohl’s study were older, with a mean age of 70 years, compared to 47 years in our study, and cognitive abilities tend to decline with age (Hedden and Gabrieli, 2004). Moreover, the participants in Pohl’s study were not in a rehabilitation clinic and, presumably, did not receive any additional therapies. It would be interesting to test both methods in the other respective diagnostic areas to better understand the effectiveness of the methods. In view of the short intervention period in our study, it is remarkable that small improvements in the MoCA were observed in both groups, particularly from pre- to post-intervention. These effects may partly be due to a practice effect in the assessment and partly attributable to the increased exercise intensity of physical activity over the four-week period.

With regards to the area of attention, the present findings appear to be in contrast with some of the literature. It has been shown that listening to music alone can improve attention in stroke patients more effectively than listening to an audio book combined with standard care (Särkämö and Soto, 2012). Studies using imaging techniques further indicate that listening to music activates neural networks involved in attention (Särkämö and Soto, 2012; Sridharan et al., 2007). These studies clearly demonstrate a connection between music and attention. In our study, we observed differential effects for the TAP subtests used for measurement of attention. Only the TAP Alertness without warning signal showed significant effects within the MBGE participants from pre- to follow-up assessment. The values at the post timepoint differed only marginally from those at the follow-up timepoint, but failed to reach significance in the *post-hoc* testing. In this task, critical stimuli are presented in a relatively dense and predictable sequence, requiring the maintenance of reaction readiness over a prolonged period (intrinsic alertness) (Sturm et al., 1999). This maintenance may have been stimulated by the therapist’s provision of varying cues for musical interaction. In contrast, the other TAP subtests showed no treatment effect. A possible explanation may lie in the higher complexity of the other TAP subtests, especially regarding the processing of visual information prior to reaction, which was not part of neither therapy regime. In terms of dual-task skills, the use of music is recommended as one of the methods (Gardiner and Thaut, 2014), but improving these skills generally seems to be difficult (Loetscher et al., 2019). However, the setting in our study was much more complex, comprising the active integration of physical, auditory and visual components. The complex nature of the MBGE stimulus landscape may therefore have distracted participants, reducing the potential impact on attention as well as dual-task capabilities.

Cognitive abilities, in particular the ability to form new episodic memories, to process information quickly and to invoke executive processes, decline throughout life (Hedden and Gabrieli, 2004). One possible confound of the present study therefore arises from the near significant group difference in age at T1. Thus, the MBGE group was on median 8 years older than the ST group with an MWU just failing to reach significance. However, the age-adjusted analysis confirmed the absence of group differences in the cognitive parameters. Therefore, the fact that patients were younger in one group than the other is an unlikely explanation for our findings.

### Effects of mood and personal preference

It was expected that the mood in the MBGE group would be higher than in the ST group, but the MDMQ showed no significant difference between the groups in any domain. It has already been shown that the combination of physical exercise and musical expression can improve mood in healthy people (Fritz et al., 2013). However, this has not been confirmed in people with dementia (Strong et al., 2022). Music-based interventions appear to have a positive effect on mood in stroke patients (Raglio et al., 2015), but this has not been clearly demonstrated for listening to music in a therapeutic context (Sihvonen et al., 2020; Baylan et al., 2016). On the whole, however, the mood scores of the participants were already in a good range at T1. This was not necessarily to be expected, because patients after a stroke often experience emotional stress, they are in a phase where they have to come to terms with their own limitations, and fears about the future may arise (McCurley et al., 2019).

One factor that may have affected the results of the study was the selection of music. Due to technical reasons, the selection was limited to a few instrumental pieces, so it was not possible to accommodate personal preferences. Possibly, participants did not fully identify with the music and therefore did not immerse themselves in the task. Indeed, participants commented during the training that the music was rather monotonous and lacked variety. The result stands in contrast to the findings that personal musical taste, such as a preference for certain styles of music, is not a decisive factor in this musical feedback intervention (Fritz et al., 2016). On the other hand there is evidence in the literature that self-selection of music can have a positive effect on visual attention in neglect (Särkämö and Soto, 2012; Chen et al., 2013) as well as vocal music has an advantage over instrumental music (Sihvonen et al., 2020). It is also thought that vocal music can increase vigilance and arousal, which is probably related to the emotional aspects of vocal music (Weiss et al., 2012). It would be interesting to investigate whether the results would have been different if the choice of music had been wider, including the preferred genre and less instrumental.

The use of group training is common in neurological contexts, frequently as a means for increasing treatment density and optimize resources (Hansen et al., 2021). Nearly 2/3 of the MBGE participants and half of the ST group prefer group training. The most common reported reasons were that group training is more fun and more motivating. Individual training, however, is also seen as an advantage because it allows for greater personal focus, more intensive practice, and fewer distractions.

In general, it is essential to consider participants’ motivation and preferences. Therefore, a separate analysis was conducted to examine whether outcomes varied depending on whether participants were assigned to their preferred training format. It was hypothesized that motivation might decrease if participants were placed in a non-preferred training constellation. However, this assumption could not be confirmed in our data. None of the comparisons diverged from the main analysis, except for the subscale “*good/bad mood*” which showed significantly different results among participants who were in their preferred constellation at follow-up.

### Effects on activities of daily living capability

The improvement of ADLs, which are closely related to cognitive impairments (Wilson et al., 2021), is a crucial aspect of neurorehabilitation, with physical training playing a key role in enhancing ADL functions (Veerbeek et al., 2014). In terms of ADL capabilities, MBGE was not found to be superior to ST. Two other studies conducted in subacute patients also showed that playing musical instruments in a therapeutic context failed to improve ADL skills (Jun et al., 2013; Grau-Sánchez et al., 2021). However, the patients in our study are already in the chronic stage, where it takes more time to achieve improvements (Schrader et al., 2024). It was found that participants of both groups improved significantly, they were able to improve from “severe” difficulties to “slight” difficulties at T2 (B-ADL cut of 5.1 points). These skills remained stable over the study period and improved further at T3, probably due the continuing rehabilitation process.

### Limitations of the study

The requirement to assign participants from the same shared flat to different groups may represent a limitation of the study. The initial plan was to form groups of participants throughout the rehabilitation center. However, due to COVID-19 pandemic restrictions, group therapy could only be conducted among individuals residing together on the same ward. This arrangement may have influenced the group atmosphere, potentially in both positive and negative ways, as participants were already familiar with one another. This may have influenced the mood of the participants during the interventions.

A further limitation was related to the scheduling of cognitive assessments, which occurred at varying times of the day alongside conventional therapy sessions. Consequently, differences in participants’ workload at these times could not be ruled out as a confounding factor. In selecting the assessments, care was taken to limit the total assessment time to a maximum of one hour to account for the patients’ endurance. Therefore, more extensive assessments that might have covered additional cognitive domains beyond those assessed by the MoCA, and thus allowed for more differentiated conclusions, were not employed.

Another limitation concerns the difficult-to-differentiate effects in the MBGE group. The MBGE group included not only the physical exercise component but also music and social components, whereas the control group received only the physical exercise component. Therefore, it is not possible to clearly determine whether the observed effects within the MBGE group are attributable to the music or the social component. However, as most improvements were observed within both groups, it seems reasonable to assume that the effects are attributable to the physical exercise component. To better elucidate the mechanisms of action of the individual factors, a larger sample size would be necessary.

Furthermore, a limitation lies in the very heterogeneous sample. It included, among others, patients with TBI as well as those with right- or left-hemisphere stroke, individuals with aphasia, visual neglect, or functional impairments alike. This complicates the interpretation and generalizability of the results, especially since not all participants had the same prerequisites for the assessments. Furthermore, all patients received additional therapies in the regular rehabilitation context, which certainly had an impact on cognition, mood, and ADLs. Therefore, the observed improvements cannot be exclusively attributed to the individual interventions.

## Conclusion

The use of music-based group therapy was not found to be superior to standard exercises in individual constellations in the areas of cognition, ADL and mood. However, both approaches demonstrated similar potential to positively influence these areas. Group therapy is associated with greater enjoyment, whereas individual therapy is associated with better concentration and more intensive training. Generally, individual preferences for group or single constellation should be considered. Further studies are needed to strengthen the evidence base for music-assisted therapy that addresses cognition and mood in people with ABI.

## Data Availability

The raw data supporting the conclusions of this article will be made available by the authors, without undue reservation.
