# Supplementary figures and images for: Effects of music-based group exercise in patients with acquired brain injury—a randomized controlled trial
Source: Front Psychol. 2026 Feb 3;16:1650872. doi: 10.3389/fpsyg.2025.1650872 (PMC12910475; doi:10.3389/fpsyg.2025.1650872)

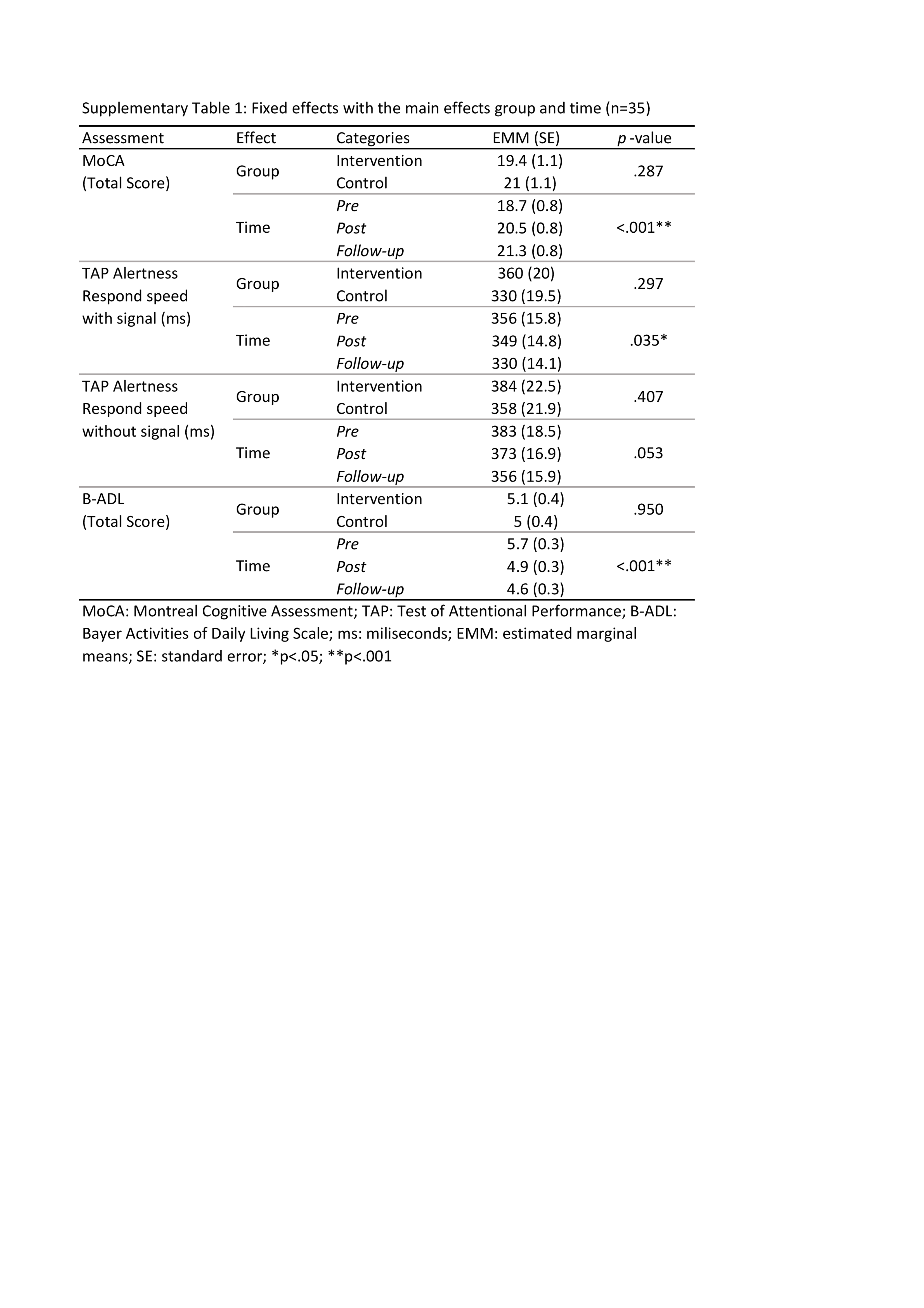

Supplement: Supplementary file 1 [file Supplementary_file_1.tiff]

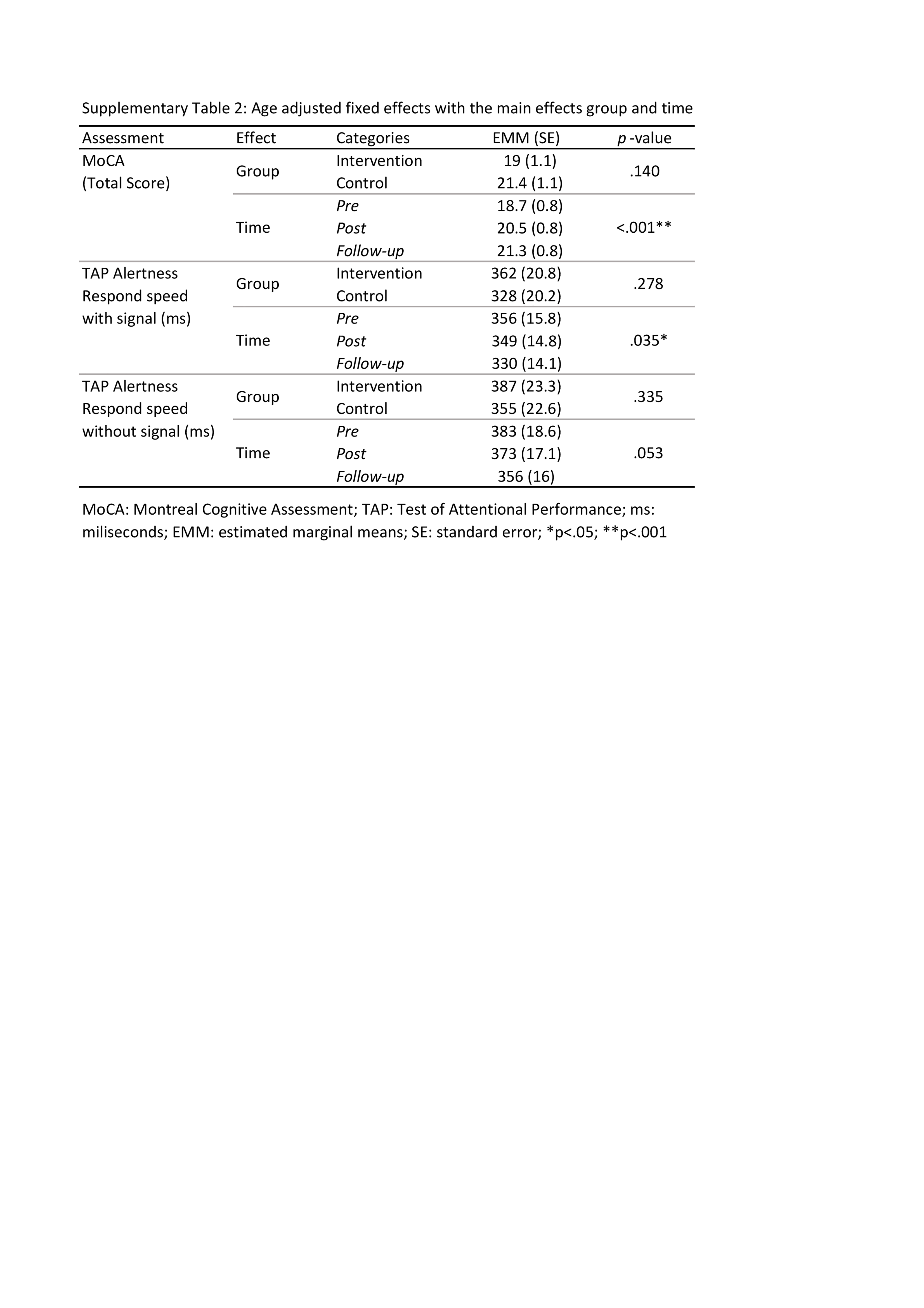

Supplement: Supplementary file 2 [file Supplementary_file_2.tiff]

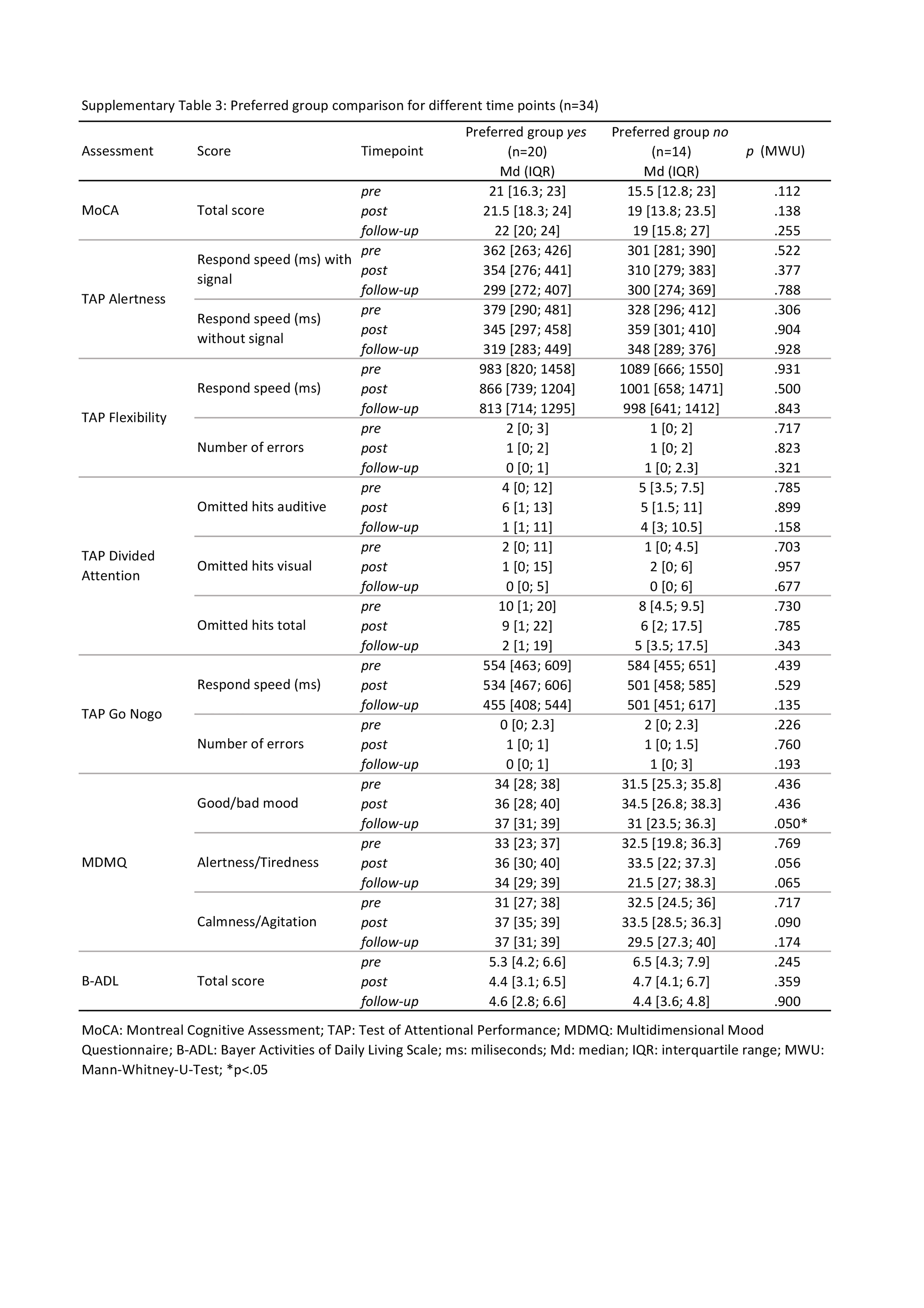

Supplement: Supplementary file 3 [file Supplementary_file_3.tiff]

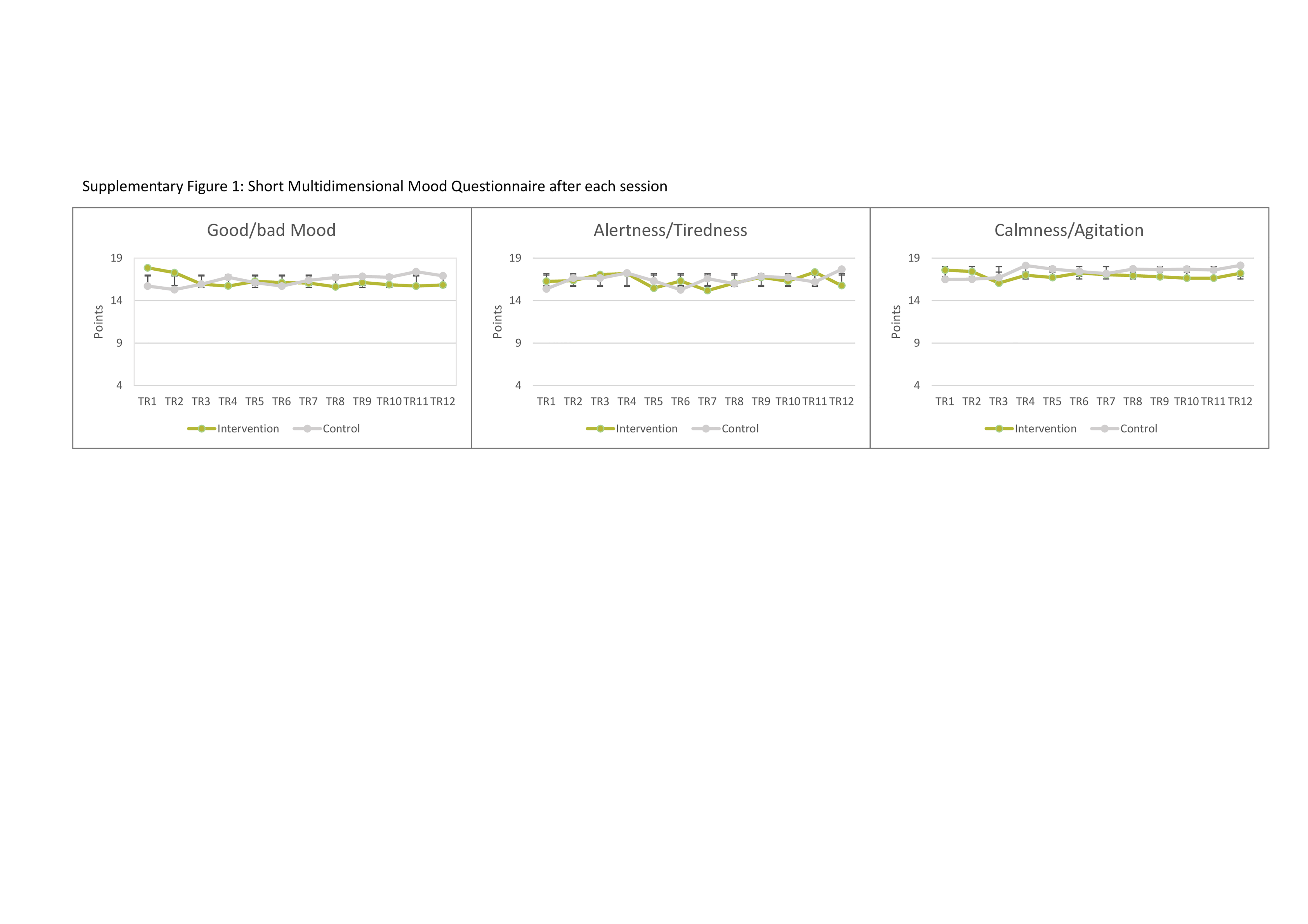

Supplement: Supplementary file 4 [file Image_1.tiff]
